# ADP Ribosylation by PARP-1 Suppresses HOXB7 Transcriptional Activity

**DOI:** 10.1371/journal.pone.0040644

**Published:** 2012-07-23

**Authors:** Xinyan Wu, Stephan Ellmann, Ethel Rubin, Minchan Gil, Kideok Jin, Liangfeng Han, Hexin Chen, Erika M. Kwon, Jianhui Guo, Hyo Chol Ha, Saraswati Sukumar

**Affiliations:** 1 Department of Oncology, Johns Hopkins University School of Medicine, Baltimore, Maryland, United States of America; 2 Department of Biochemistry and Molecular & Cellular Biology, Georgetown University Medical Center, Washington, D.C., United States of America; French National Centre for Scientific Research, France

## Abstract

Interactions with cofactors regulate transcriptional activity and also help HOX proteins to achieve the specificity required for transcriptional regulation of target genes. In this study, we describe a novel protein/protein interaction of HOXB7 with poly (ADP-ribose) polymerase-1 (PARP-1) that involves the homeodomain of HOXB7 and the first zinc finger domain of PARP-1. Upon binding to PARP-1, HOXB7 undergoes poly(ADP-ribosyl)altion resulting in a reduction of its transcriptional activity. Since aspartic acid and glutamic acid residues are acceptors of the ADP ribose moiety transferred by PARP-1, deletion of the evolutionarily conserved C-terminal Glu-rich tail of HOXB7 dramatically attenuates ADP-ribosylation of HOXB7 by PARP-1. Further, a mutant of HOXB7 without the Glu-rich tail loses the ability to be negatively regulated by PARP-1 and becomes transcriptionally more active in luciferase reporter assays. Since the homeodomain is highly conserved among HOX proteins, five other HOX proteins were tested. All six showed interaction with, and were poly(ADP-ribosyl)ated by PARP-1. However, among them, this modification altered the DNA binding activity of only HOXA7 and HOXB7. In summary, this study identifies a new interacting partner of HOX proteins. More importantly, this study reveals a novel mechanism whereby polyADP-ribosylation regulates transcriptional activities of HOX proteins such as HOXB7 and HOXA7.

## Introduction

The HOX family of proteins is composed of 39 evolutionarily conserved transcription factors characterized by a 61-amino acid DNA binding domain (homeodomain). The 39 HOX genes are organized into four paralogous clusters, HOX-A to -D, on four different autosomal chromosomes, and are colinearly expressed during embryogenesis in the order of their genomic localization [1]. The strict temporal and spatial expression pattern of HOX genes is critical for HOX protein regulation of embryonic development and for the maintenance of homeostasis in adulthood. Deregulation of their expression has been increasingly correlated with a variety of diseases including cancers [2]. For example, over-expression of HOXA9 in mouse progenitor cells leads to myeloid leukemia [3], [4]. HOXB7 mRNA is overexpressed in breast and ovarian carcinomas and can promote metastasis by induction of epithelial-mesenchymal transition (EMT) [5]; HOXB13 overexpression correlates with tamoxifen resistance in breast cancer [6], [7]. Loss of expression of HOX proteins is also found to be related to tumorigenesis. For instance, HOXA5 expression is frequently lost in high-grade breast tumors and leads to resistance to apoptosis [8], [9].

Despite decades of research on HOX genes, their downstream target genes still remain poorly defined. The complexity of the regulatory networks controlled by HOX proteins, added to their short consensus-binding motif has hampered identification of their target genes. Because of the high homology of the homeodomain and simplicity of the consensus core motif (TAAT) [10], it is likely that interaction partners of HOX proteins and post-translational modification of HOX proteins play a key role in regulating their transcriptional activity to assist HOX proteins attain their functional specificity. Interactions between HOX proteins and PBX1 [11], [12], [13] or CBP/P300 [14], and more recently SMAD proteins [15], [16] alter the DNA-binding ability of HOX proteins and either increase or suppress their transcriptional activities. Conversely, under some circumstances, HOX proteins also manipulate the functions of their partners. HOX proteins block the histone acetyltransferase activity of CBP [14], and as recently shown by our group [17], HOXB7 binds to both DNA-PK complex and PARP-1. The interaction between HOXB7 and DNA-PK increased the efficiency of DNA double strand break repair activity [17]. However, the interaction between HOXB7 and PARP-1 and the biological function of this interaction was not addressed.

PARP-1 is an abundant chromatin-associated enzyme in the cell that post-translationally modifies proteins via poly(ADP-ribosyl)ation, and has a demonstrated involvement in multiple crucial cellular processes including DNA replication, DNA repair, apoptosis, and genomic stability [18]. In addition, PARP-1 also regulates gene expression at two different levels [19]. First, PARP-1 modulates the epigenome by modifying histones, or by regulating DNA methylation status to alter chromatin structure. Second, PARP-1 regulates gene expression by forming complexes with other transcription factors. Focusing on the latter, a growing body of work has shown many functional interactions between PARP-1 and transcriptional factors. Alternatively, PARP-1 functions as a co-activator by interacting with B-MYB [20], [21], HLTV TAX [22], or HIF1 [23] and increases their DNA-binding activity. On the other hand, PARP-1 interacts with proteins like YY1 [24] to repress their transcriptional activity. Furthermore, PARP-1 enzymatic activity is required for transcription regulation in some contexts, but dispensable in others. For example, PARP-1 catalytic activity is indispensable for regulating gene expression in cooperation with TEF-1 [25] or with topoisomerase II in ERα-dependent gene activation [26], and in regulating the expression of CXCL1 [27]. On the contrary, as a component of a transcriptional complex with B-MYB [21] or RARα/RXRα heterodimers [28], PARP-1 does not need its catalytic domain to enhance transcription. Of note, when PARP-1 functions as a transcriptional repressor, PARP-1 can poly(ADP-ribosyl)ate its interacting transcription factors YY1, NF-κB, and TBP, and inhibit their binding to DNA *in vitro* [reviewed by Kraus [29]].

In this study, we show that PARP-1 binds to HOXB7 and to other HOX proteins, and can poly (ADP-ribosyl)ate HOX proteins both *in vivo* and *in vitro*. However, this modification inhibits the transcriptional activity of only HOXB7 and HOXA7, but not of several other HOX proteins, by decreasing their DNA-binding potential. We provide evidence that PARP-1 enzymatic activity is necessary for its transcription repressive effect on HOXB7 and HOXA7. This study demonstrates the interaction between PARP-1 and HOX proteins, and suggests a potential function for PARP-1 in embryonic development and maintenance of homeostasis in adulthood via regulation of transcriptional activity of HOX proteins.

## Results

### Interaction between HOXB7 and PARP-1

**Figure 1:**
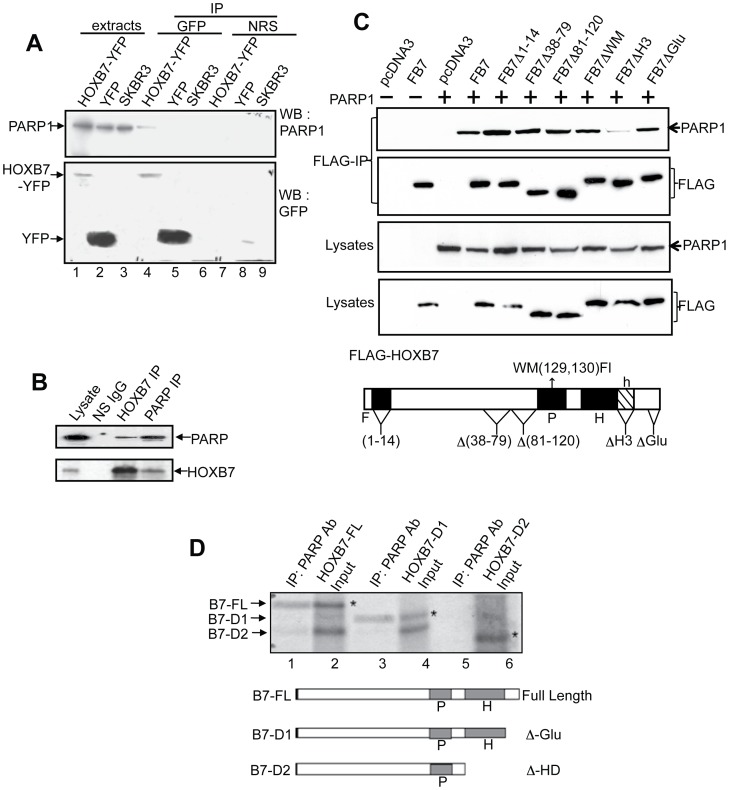
HOXB7 interacts with PARP-1. A. Co-immunoprecipitation of PARP-1 with HOXB7-YFP in SKBR3 cells. SKBR3 cells were stably transfected with HOXB7-YFP (lanes 1, 4, and 7) or YFP alone as a vector control (lanes 2, 5, and 8) prior to immunoprecipitation with GFP antibodies and subsequent Western blotting of precipitated proteins. Parental SKBR3 cells, which lack detectable HOXB7, were used as controls as well (lanes 3, 6, and 9). Lanes 1 to 3, protein levels in 100µg of total cell extract (5% of input); lanes 4 to 6, proteins that precipitated with HOXB7-YFP or controls that did not express HOXB7 (SKBR3-YFP and parental cells). Normal rabbit serum (NRS) was used to control for specificity (lanes 7–9). B. Endogenous interaction between HOXB7 and PARP-1. Extracts of MCF-7 cells were co-immunoprecipitated with antibodies to PARP-1, HOXB7 or p53 as a nonspecific IgG (NS IgG). Subsequent immunoblotting was done with the antibodies indicated. C. Flag-tagged HOXB7 or constructs in which select regions were deleted or mutated, were transiently transfected into CHO cells together with a PARP expression construct (PARPpCR3.1, where indicated, empty plasmid was used as control) to determine if a specific region of HOXB7 mediated its interaction with PARP. Co-immunoprecipitation with FLAG antibodies (top panel) was performed followed by immunoblot with PARP antibodies. The lower panel shows protein expression of all transfected plasmids. Structure of FLAG-HOXB7 showing locations of point mutations and deletions is shown below. F: Flag tagged HOXB7, P: pentapeptide, H: homeodomain, h: helix 3 of the homeodomain. D. Full length HOXB7 or deletion constructs B7-D1 or B7-D2 were transcribed and translated *in vitro* in the presence of ^35^S-methionine prior to mixing with *in vitro* transcribed and translated PARP-1. Immunoprecipitation was performed with PARP-1 monoclonal antibodies (lanes 1, 3 and 5). Complexes were resolved by SDS-PAGE and subjected to autoradiography for 24 hours. Lanes 2, 4 and 6 are input (20%) from the TNT reactions. Deletions D1 and D2 of full length HOXB7 are shown in Figure 2. Asterisks point to the specific bands for HOXB7 full-length or deletion proteins. P: pentapeptide, H: homeodomain.

By using GST-HOXB7-Sepharose affinity chromatography with cell extracts from several breast epithelial cells, both normal (MCF10A, MCF12A) and cancer (SKBR3), we identified PARP-1 as a HOXB7-interaction partner [17]. To confirm this finding, anti-GFP polyclonal antibody (that can also recognize YFP) was used for co-immunoprecipitation to pull down the YFP-HOXB7 fusion protein transfected into SKBR3 cells, followed by Western blot analysis. As shown in Figure 1A, PARP-1 protein specifically co-immunoprecipitated with the YFP-HOXB7 fusion protein, but not with YFP control protein, or following co-IP with a nonspecific control antibody. To test the interaction under physiological conditions, MCF-7 breast cancer cells that endogenously express both HOXB7 and PARP-1 were used. In MCF-7 cell extracts, co-immunoprecipitation was performed using monoclonal PARP-1 or HOXB7 antibodies. As shown in Figure 1B, either PARP-1 or HOXB7 antibodies can reciprocally co-immunoprecipitate the endogenous HOXB7 and PARP-1 complex. Thus, the data is supportive of an interaction between endogenous HOXB7 and PARP-1 proteins in breast epithelial cells.

**Figure 2:**
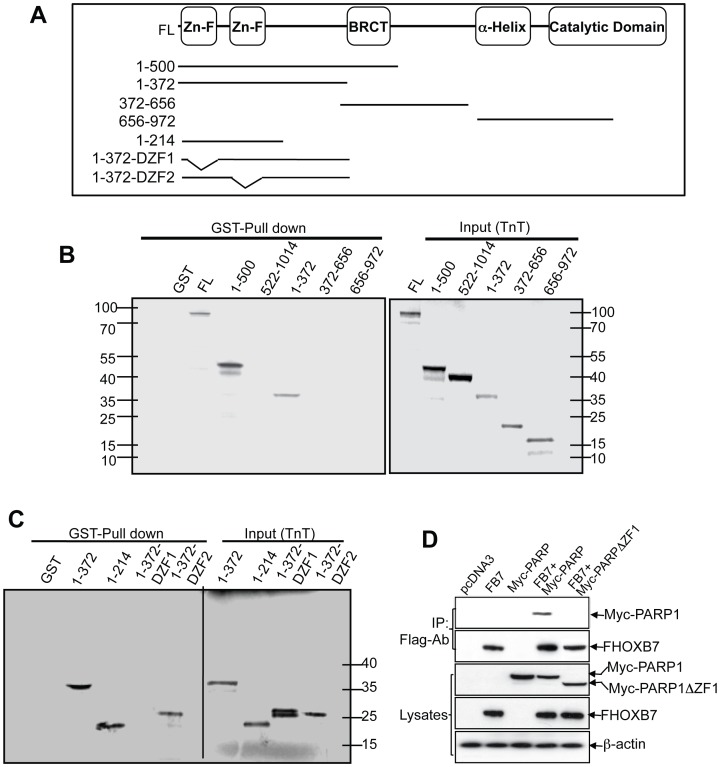
Defining regions of PARP-1 that interact with HOXB7. A. The molecular structure of PARP-1 and the deletion constructs used in the present study are shown in the panel. B, C. Full-length PARP-1 and different truncations of PARP-1 proteins as indicated were *in vitro* transcribed and translated (TNT) in presence of the ^35^S-methionine and subjected to GST-HOXB7 or GST pull-down assay (left panels Fig. 2B and 2C). The right panels of Figure 2B and 2C show the input (20%) of the PARP-1 full-length and truncation proteins from TNT products. D. Plasmids coding Flag-tagged HOXB7 and Myc-tagged wild type PARP1 or first zinc-finger deleted PARP1 were cotransfected into MCF-7 cells. Cell lysates were immunoprecipitated with anti-Flag antibody. PARP-1 and HOXB7 protein were detected with anti-Myc or anti-Flag antibodies.

### The Homeodomain Mediates the Interaction between HOXB7 and PARP-1

It was of interest to define the domains of HOXB7 involved in the interaction with PARP-1. We constructed a number of HOXB7 deletions in which distinct regions of HOXB7 were removed, and, in addition, used previously described [30] deletion constructs and HOXB7 mutants. To render them convenient for detection and immunoprecipitation, all HOXB7 mutants were constructed in-frame with the FLAG-tag coding sequence. These mutants included: 1) mutations in the PBX1-binding region [FB7WM (W129F, M130I)], shown to be required for high affinity DNA-binding and transactivation; 2) deletion of the glutamic acid-rich carboxyl terminal tail (FB7ΔGLU), or 3) deletion of helix 3 of the homeodomain (FB7Δh3). In addition, mutants of the FLAG-tagged HOXB7 expression construct were tested which had: 4) deletions of amino acids 1–14 (FB7Δ1-14), 5) amino acids 38-79 (FB7Δ38-79) or, 6) 81-120 (FB7Δ81-120). Transient transfection of these constructs into CHO cells together with PARP-1 showed that, with the exception of FB7Δh3 (helix 3 deletion) (Fig. 1C, lane 4) that showed reduced PARP-1 binding, other deletions/mutations in HOXB7 had no effect on PARP-1 binding to HOXB7. These findings suggested that the homeodomain of HOXB7 might mediate its interaction with PARP-1. Further confirmation was sought. Immunoprecipitation of PARP-1 with *in vitro* transcribed and translated HOXB7 and its deletion mutants showed that HOXB7 utilized its homeodomain for interaction with PARP-1 (Fig. 1D). While the full-length and glutamic acid tail-deleted forms of HOXB7 still bound to PARP-1, deleting the entire homeodomain in HOXB7 completely abolished the interaction. These findings established a critical function for the HOXB7 homeodomain in mediating interactions with PARP-1.

### The First Zinc Finger Domain in PARP-1 is Indispensible for the Interaction

Next, to identify the HOXB7 interaction domain in the PARP-1 protein, a GST pull-down assay was performed. Sepharose-immobilized GST-HOXB7 fusion protein was used to precipitate *in vitro* transcribed and translated full-length PARP-1 and truncated mutant proteins that span the entire PARP-1 protein including the zinc finger domains, auto-modification domain, and catalytic domain (Fig. 2A). A first round of screening showed that the C-terminal moiety of PARP-1 from 372 to 1014 did not interact with HOXB7, but that the N-terminal part (1-372) mediated the interaction (Fig. 2B). This 372 amino acid peptide contains two zinc finger domains which recognize DNA strand breaks and bind to DNA, and the cleavage site at which PARP-1 is cleaved into two peptides (24 kD and 89 kD) during apoptosis [18]. In order to define the interaction domain, we made constructs by deleting either the first (1-372ΔZF1), or the second zinc finger domain (1-372ΔZF2) from the PARP-1 N-terminal moiety (1-372), and the N-terminal cleavage domain (1-214). The GST-pull-down assay revealed that GST-HOXB7 bound to all three peptides that contained the first zinc finger domain (1-372, 1-372ΔZF2, and 1-214), but not to the peptide lacking the first zinc finger domain (1-372ΔZF1) (Fig. 2C). We further confirmed that the first zinc figure domain is required for HOXB7-PARP-1 interaction by co-transfection of plasmids of Flag-HOXB7 and PARP-1 with deletion of just the first zinc finger, into MCF-7 cells. The Flag-tag antibody based co-immunoprecipitation showed that deletion of first zinc finger in PARP1 disrupted the protein/protein interaction (Fig. 2D). These data indicated that the first zinc finger domain directly bound to HOXB7. Thus, the results of this series of experiments showed that the PARP-1-HOXB7 interaction occurs through the first zinc finger of PARP-1 and the homeodomain of HOXB7.

### Poly(ADP-ribosyl)ation of HOX Proteins by PARP-1

**Figure 3:**
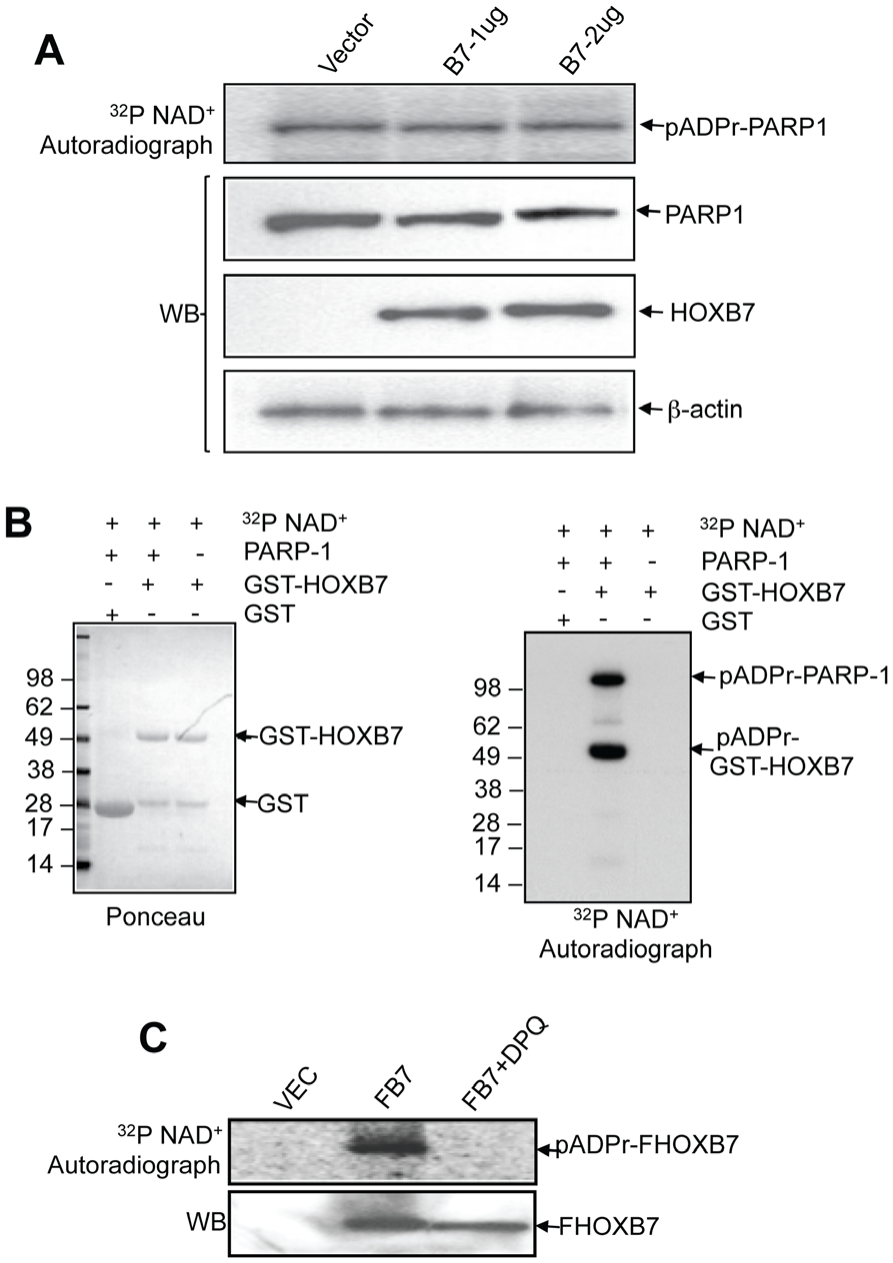
HOXB7 is the substrate of PARP-1 and is poly(ADP ribosyl)ated by PARP-1. A. Different amounts of Flag tagged HOXB7 plasmids were transfected into SKBR3 cells, cells were harvested and permeabilized by 0.01% digitonin in PARP-1 activity reaction buffer. PARP-1 auto-modification was visualized by autoradiograph after the cell lysates were separated by SDS-PAGE (top panel). Immunoblot with anti-Flag, PARP-1 and β-actin antibodies were performed after autoradiography (bottom panels). B. GST and GST-HOXB7 fusion proteins were incubated with or without purified PARP-1 protein or ^32^P NAD+. After 30 minutes incubation, free 32P NAD+ and unbound PARP-1 proteins were washed off with reaction buffer. Proteins on glutathione sepharose beads were then separated by SDS-PAGE and transferred to PVDF membrane and stained with Ponceau (left panel). The poly(ADP ribosyl)ated proteins were visualized by autoradiography (right panel). C. Vector control and Flag-tagged HOXB7 plasmids were transfected into SKBR3 cells. Cells were harvested and incubated with PARP-1 activity assay buffer including ^32^P NAD+ or PARP-1 inhibitor (20 µM DPQ). Cell lysates were immunoprecipitated with anti-Flag antibody and separated by SDS-PAGE followed by autoradiography (top panel) and immunoblotting with anti-Flag antibodies (bottom panel).

To explore the functional consequences of this interaction, we first examined the enzyme activity of PARP-1. PARP-1 uses NAD+ as a substrate and transfers an ADP-ribose moiety to acceptor proteins, including itself; this automodification is the major catalytic action of PARP-1 [19]. We examined PARP-1 automodification activity by adding ^32^P-labeled NAD+ into permeabilized SKBR3 cells that were transiently transfected with different amounts of HOXB7-expressing plasmids, followed by separation of the proteins by SDS/PAGE. As shown in the top panel of Figure 3A, the automodification levels of PARP-1 showed no significant change with increasing expression levels of HOXB7 (Fig. 3A). Given that PARP-1 can also modify many other DNA associated proteins, HOXB7 may also be poly(ADP-ribosyl)ated via interaction with PARP-1. To test this possibility, we incubated GST or GST-HOXB7 fusion proteins and ^32^P-NAD+ with or without PARP-1 protein. GST-HOXB7 specifically modified only when the enzyme PARP-1, substrate ^32^P-NAD+ and GST-HOXB7 were combined together. The GST protein by itself was not modified (Fig. 3B). The modification on HOXB7 was examined in intact cells as well. FLAG-tagged HOXB7 (FB7)-transfected SKBR3 cells were permeabilized with 0.01% digitonin and incubated with ^32^P-NAD+ followed by lysis and immunoprecipitation with anti-FLAG antibody. The autoradiograph showed the presence of ^32^P poly(ADP-ribosyl)ated FB7; this reaction could be suppressed by the PARP inhibitor, DPQ (Fig. 3C). These results suggested that HOXB7 is, in all likelihood, poly(ADP-ribosyl)ated via interaction with PARP-1.

### PARP-1 Suppresses HOXB7 Transcription Activity by polyADP-ribosylation

**Figure 4:**
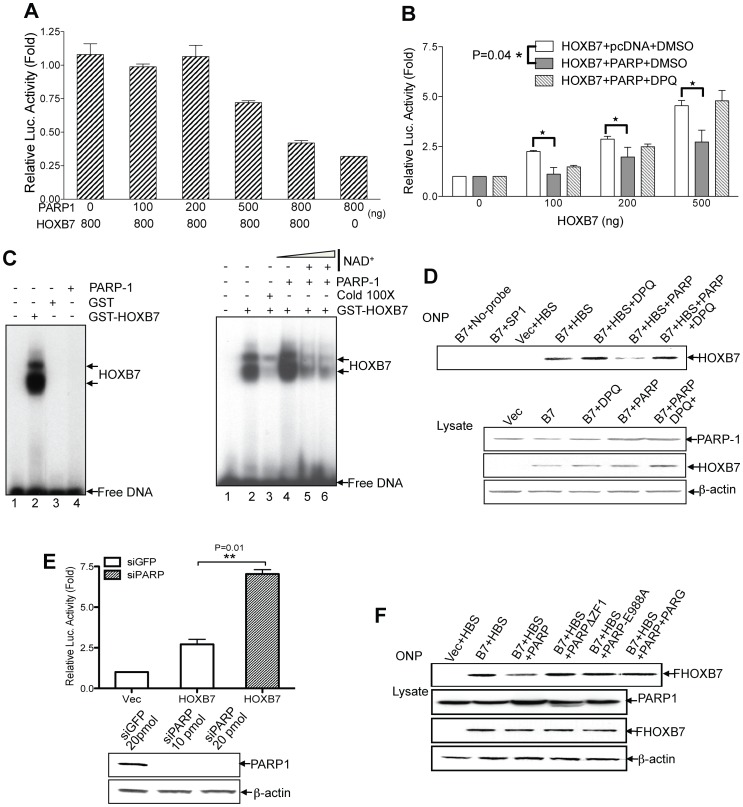
Poly(ADP ribosyl)ation on HOXB7 by PARP-1 reduces HOXB7 transcriptional activity. A. Luciferase reporter assay were performed 24 hours after transfection of HOXB7, different amounts of PARP-1 plasmid as indicated, along with TTATpGL3 reporting reporter plasmid containing HOXB7 binding motifs, into HeLa cells. B. Different amounts of HOXB7 plasmids as indicated, with or without 400 ng PARP-1 plasmid and/or its inhibitor (10 nM DPQ), were transfected into HeLa cells, and luciferase activity assay was performed 24 hours after transfection. Paired student t-test was applied to compare the differences between both groups with or without the overexpression of PARP-1. C. Left panel: GST or GST-HOXB7 proteins were incubated with ^32^P labeled oligonucleotide consisting of the HOXB7-consensus binding sequence (HBS) in presence or absence of PARP-1 proteins. Right panel: GST-HOXB7 proteins were incubated with ^32^P labeled oligonucleotide consisting of the HOXB7-consensus binding sequence (lane 2) or in combination with 100-fold excess of unlabeled oligonucleotide (lane 3), or purified PARP-1 protein (lane 4), or purified PARP-1 and increasing amounts of NAD+ (lane 5, 6). D. Oligonucleotide precipitation assays (ONP) were performed by using SKBR3 cells transfected with vector, Flag HOXB7 and/or PARP-1 plasmids. One group of HOXB7 and PARP-1 transfectants were treated with DPQ 6 hours after transfection. Cell lysates were incubated with biotinylated HBS and/or DPQ. DNA protein complexes immobilized with streptavidin agarose beads were subjected to western blot with Flag antibody. Biotinylated SP1 binding site probe was used as negative control. Cell lysates were also blotted with anti-Flag, PARP-1 or β-actin antibody as loading control. E. Top panel: HOXB7 expressing and TTATpGL3 reporting plasmids were co-transfected with siPARP or siGFP control oligomer into HeLa cells, and luciferase activity was measured 24-hours post transfection. P value, derived from the Student t-test is shown. The efficacy of siPARP oligomer was tested by transient transfection of siRNA into HeLa cells and evaluated by western blot (bottom panel). F. Flag-HOXB7 plasmids were co-transfected with PARP-1 full-length or PARP-1 mutant plasmids, as indicated, into SKBR3 cells. Cell lysates were used for ONP assay in presence or absence of poly ADP ribose glycohydrolase (PARG) (top panel). The cell lysates were also used for western blot with anti-Flag, PARP-1 or β-actin antibody as loading control.

As an abundant nuclear protein, PARP-1 can modify a wide spectrum of proteins, the majority of which are DNA-associated proteins. A battery of studies has shown that PARP-1 can poly(ADP-ribosyl)ate chromatin-associated proteins such as histones, and disrupt compact chromosome structures to facilitate transcription [29]. PARP-1 can also poly(ADP-ribosyl)ate transcription factors (SRY, YY1 and TBP and others) [29], [31], resulting in a massive increase of negative charge on these transcription factors which, as a consequence, decreases their DNA-binding ability. We hypothesized that PARP-1 mediated modification may also alter HOXB7-mediated transcriptional function. To test this, luciferase reporter assays were performed. Different amounts of HOXB7 and PARP-1 were cotransfected with a luciferase reporter construct containing 6 HOX-responsive elements (TTAT) [11] into HeLa cells. Increasing the concentration of PARP-1 resulted in a stepwise decrease in transcriptional activation of the luciferase reporter by HOXB7 (Fig. 4A). In a second set of experiments, we kept the amount of PARP-1 constant while increasing the amount of HOXB7 (Fig. 4B). HOXB7 transcriptional activity was always lower in cells that had been transfected with the PARP-1 expression plasmid, in comparison to cells transfected with control vector. Moreover, treatment with the PARP inhibitor, DPQ (3,4-Dihydro-5[4-(1-piperindinyl)butoxy]-1(2H)-isoquinoline), restored the activity of HOXB7 on the reporter even in the presence of PARP-1 (Fig. 4B). To determine specificity of the transcriptional suppression of HOXB7 by PARP-1, the SMAD3/4 responsive luciferase report system was used as a control. As predicted, overexpression of PARP-1 did not interfere with the transcriptional activity of SMAD3/4 (data not shown). Thus, PARP-1 mediated modification appears to reduce HOXB7-mediated transcriptional function.

Next, to test if the suppression of transcriptional activity of HOXB7 by PARP-1 is the result of the interaction between PARP-1 and HOXB7 or the poly(ADP-ribosyl)ation of HOXB7 itself but not other HOXB7 interacting cofactors such as PBX1, we performed electrophoretic mobility shift assays. The left panel of Figure 4C shows that the probe which contains HOX protein binding motifs [32] bound specifically to HOXB7 but not to GST or PARP-1. The addition of PARP-1 and substrate NAD+ significantly attenuated HOXB7-DNA-binding activity. The suppression that occurred was similar to the levels attained by competition with unlabeled probe present at concentrations 100 times higher than the ^32^P-labeled probe (Fig. 4C). Notably, addition of PARP-1 alone did not suppress the ability of HOXB7 binding (Fig. 4C) to the probe, and did not result in a band shift, suggesting that PARP-1 is not stably interacting with HOXB7 at this level. These data showed that the interaction between PARP-1 and HOXB7 alone was not sufficient to suppress HOXB7 binding to DNA. In fact, it raised the possibility that the PARP-1 enzymatic activity may be necessary for the suppression.

To further confirm the suppression of HOXB7-mediated transactivation by PARP-1, oligonucleotide precipitation (ONP) assays were performed in SBKR3 breast cancer cells. We found that, compared to the cells transfected with HOXB7 and control vector plasmids, the binding ability of HOXB7 to the probe containing homeobox binding sequence (HBS) [32] was reduced in PARP-1-overexpressing cells. Treatment with DPQ restored the binding ability (Fig. 4D). A particularly interesting finding was that DPQ treatment of cells not transfected with PARP-1 also increased DNA-binding by HOXB7 (Fig. 4D), suggesting an effect of endogenous PARP-1 on HOXB7 activity. In line with this notion, siRNA-mediated knockdown of endogenous PARP-1 elevated HOXB7 transcriptional activity in the luciferase reporter assay (Fig. 4E). The same suppression pattern was reproduced in a second breast cancer cell line, MCF-7 (data not shown).

### The First Zinc Finger Domain and Catalytic Activity of PARP-1 are Indispensible for the Suppression of HOXB7 Transcriptional Activity

In the experiments described above, we showed that the first zinc finger domain in PARP-1 mediated interaction between PARP-1 and HOXB7, and that the enzymatic activity of PARP-1 is critical for the negative regulation of HOXB7 activity. In order to study whether both the interaction of PARP-1 with HOXB7 followed by its modification are essential for the suppression effect, we created mutant forms of PARP-1 protein: 1) deletion of the first zinc finger (PARPΔZF1) that mediates HOXB7-PARP-1 interaction and, 2) E988A mutant (PARP-1-E988A) that lacks ADP-ribose polymerase activity [33]. The ONP assay showed (Fig. 4F) that both mutations, PARPΔZF1 and PARP-E988A resulted in the loss of PARP-1′s ability to suppress HOXB7 DNA binding activity. Furthermore, addition of poly(ADP-ribose) glycohydrolase (PARG) to hydrolyze the ADP-ribose polymers present on HOXB7 resulted in the restoration of HOXB7’s ability to bind DNA (Fig. 4F). These data provided strong evidence that poly(ADP-ribosyl)ation of HOXB7 is the key mechanism underlying PARP-1-mediated negative regulation of HOXB7 transcriptional activity.

### The Glutamic Acid Rich Tail of HOXB7 is a Negative Regulatory Element for HOXB7 Transactivation

**Figure 5:**
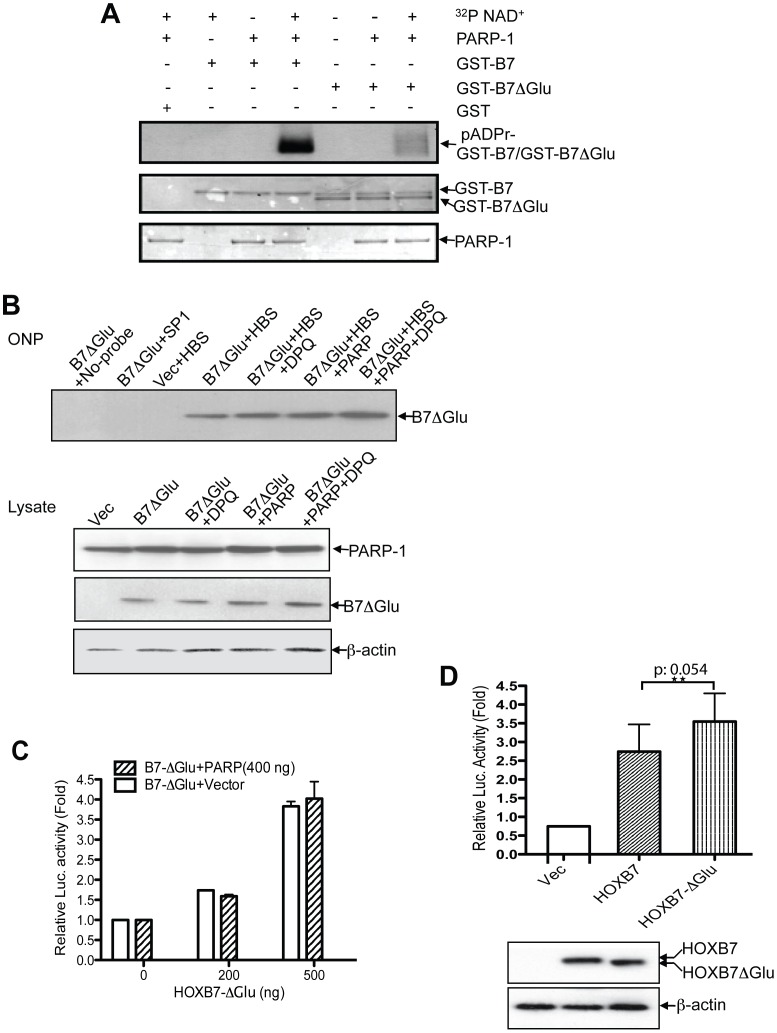
Glutamic acid-rich tail in HOXB7 is the major site Poly(ADP ribosyl)ated by PARP-1. A. GST, GST-HOXB7 and GST-HOXB7ΔGlu fusion proteins were incubated with or without PARP-1 protein or ^32^P NAD+. Proteins were then separated by SDS-PAGE and transferred to PVDF membrane and stained with Ponceau (two bottom panels). The poly(ADP ribosyl)ated proteins were visualized by autoradiograph (top panel). B. Vector or Flag HOXB7ΔGlu plasmids were transfected with or without PARP-1 plasmids into SKBR3 cells, and one set of HOXB7ΔGlu and PARP-1 co-transfected cells was treated with DPQ 6 hours post transfection. Cell lysates were used for ONP assays and western blots. C. TTATpGL3 reporting plasmids were transfected together with increasing amount of HOXB7ΔGlu, and 400 ng PARP-1 or 400 ng empty vector plasmids as control in SKBR3 cells. Luciferase activity was measured 24-hour after transfection. D. Vector, HOXB7 full-length and HOXB7ΔGlu plasmids (300ng) were each co-transfected with TTATpGL3 reporter plasmids, and luciferase activity was measured 24-hour after transfection. Student t test was performed to compare luciferase activity between groups of HOXB7 full-length and HOXB7ΔGlu transfectants. Student t test was applied to compare the differences between HOXB7 and HOXB7ΔGlu groups. Cell lysates were western blotted with anti-Flag or β-actin antibody.

Aspartic acid (Asp) and glutamic acid (Glu) residues are the known acceptors of first ADP ribose moiety transferred by PARP-1. Of note, seven out of the last 8 amino acids at the C-terminus of HOXB7 are Glu residues. We predicted that this Glu-rich tail might be the target site of ADP-ribosylation and play a key role in the suppression of HOXB7 binding and transcription by PARP-1. To test this concept, the PARP modification assay was performed with the HOXB7 glutamic acid tail-deletion construct, GST-B7-ΔGlu. The autoradiograph showed that the ADP-ribosylation level was dramatically lower in GST-B7-ΔGlu compared to the GST-HOXB7 wild type fusion protein (Fig. 5A). These results suggested that the glutamic acid rich tail in HOXB7 is the major poly(ADP-ribosyl)ation site for PARP-1, but not the only site that can be modified by PARP-1. Upon removal of the C-terminal Glu-rich tail, modification occurred nevertheless, in the N-terminus to the Glu-rich tail of HOXB7, albeit at a lower level.

To further confirm that the C-terminal Glu-rich tail of HOXB7 is important for PARP-1-mediated suppression of DNA binding ability of HOXB7, the ONP assay was performed. The data showed that, unlike the wild type HOXB7 (Figure 4D), deletion of Glu-rich tail (HOXB7ΔGlu) completely abolished the PARP-1-mediated suppression of DNA binding (Fig. 5B). This phenomenon was also observed in the promoter luciferase assay. HOXB7ΔGlu displayed similar levels of transactivation, irrespective of whether PARP-1 was overexpressed or not (Fig. 5C). We had shown in the previous experiment that siPARP significantly increases HOXB7-mediated transactivation (Fig. 4E). If the Glu-rich tail is a repressive element in HOXB7, it follows that HOXB7ΔGlu should have higher transactivation capability than the wild type HOXB7. As predicted, in comparison with wild type HOXB7, the deleted form, HOXB7ΔGlu, displayed higher transcriptional activity (Fig. 5D). Thus, in this context of PARP-1/HOXB7 interaction, the glutamic acid rich tail of HOXB7 behaves as a negative regulatory element.

### PARP-1 Suppresses the DNA Binding Ability of HOXA7 but not Other Four HOX Proteins

**Figure 6:**
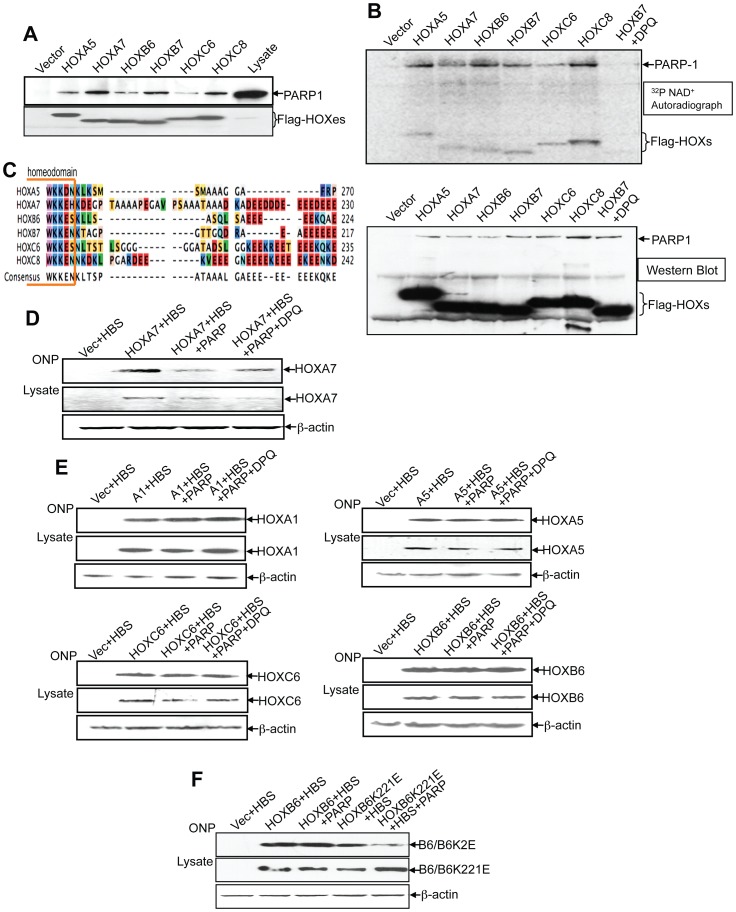
PARP-1 interacts and modifies other HOX proteins. A. SKBR3 cells were transfected with empty vector, or Flag-tagged HOXA5, B6, B7, C6 and C8, respectively. Cell lysates were immunoprecipitated with anti-Flag antibody, and western blotted with anti-PARP-1 (top panel) and Flag antibodies (bottom panel), respectively. HOXC6 transfected SKBR3 cell lysate was used as loading control. B. Vector control and Flag-tagged HOX plasmids were transfected into SKBR3 cells as indicated. Cells were harvested and incubated with PARP activity reaction buffer containing 0.01% digitonin and ^32^P NAD+. Cells were then lysed after incubation and immunoprecipitated with anti-Flag antibody. The precipitated complexes were then separated by SDS-PAGE, transferred to the nitrocellulose membrane that was used for autoradiography (top panel) and western blot (bottom panel) using anti-PARP-1 and anti-Flag antibodies. C. Multiple sequence alignment of the C-terminal peptides of HOXA5, HOXA7, HOXB6, HOXB7, HOXC6 and HOXC8 show extent of homology. D. The ONP assay was performed with the SKBR3 cells transfected with Flag HOXA7 with or without PARP-1, and one set of the HOXA7 and PARP-1 co-transfected cells was treated with DPQ 6 hours post transfection. Cell lysates were used for ONP (top panel) and western blots (bottom panel). E. ONP assays were performed with SKBR3 cells transfected with Flag HOXA1, Flag HOXA5, Flag HOXC6 or Flag HOXB6 plasmids with or without PARP-1 plasmids, and one set of each of the HOX and PARP-1 co-transfected cells was treated with DPQ 6 hours post transfection. Cell lysates were used for ONP assays (top panel) and western blots (bottom panel). F. ONP assay was performed with the SKBR3 cells transfected with Flag-tagged HOXB6 or the Lys to Glu mutant, HOXB6K221E, with or without PARP-1. Cell lysates were used for ONP assays (top panel) and western blots (bottom panel).

Based on our findings that the homeodomain mediates the interaction between HOXB7 and PARP-1, and since the homeodomain is the most conserved region among HOX proteins, it is reasonable to predict that PARP-1 will interact with other HOX proteins as well. Since the Glu-rich tail in HOXB7 is critical for the suppression of its transcriptional activity by PARP-1, we performed homology comparisons among the 39 HOX family members to narrow down our selection of HOX genes to test. We found that, like HOXB7, four other HOX proteins (HOXA7, HOXB6, HOXC6 and HOXC8) have Glu-rich tails (Fig. 6C). To test whether PARP-1 is a common partner of HOX proteins and can also suppress transcriptional regulation mediated by these other HOX proteins, the coding sequences of the four HOX proteins containing Glu-rich tails (HOXA7, HOXB6, HOXC6 and HOXC8) and a HOX protein without Glu-rich tails (HOXA5) were cloned into FLAG-tag expression vector. By immunoprecipitation with the anti-FLAG antibody, as predicted, we found that PARP-1 is a common binding partner as well for all five HOX proteins tested (Fig. 6A). The PARP-1 modification assay showed that each of these HOX proteins was a substrate of PARP-1 (Fig. 6B), and the PARP enzymatic inhibitor, DPQ, significantly suppressed their poly(ADP-ribosyl)ation. This finding is in line with our previous data (Fig. 5A) that although the Glu-rich tail is the major modification site, there are other Glu or Asp residues in HOXB7 protein that can be ADP-ribosylated by PARP-1. This also provides an explanation for why HOXA5, which lacks the C-terminal Glu tail, could still be modified by PARP-1.

Next, to address whether PARP-1 can also suppress the DNA binding ability of other HOX proteins, HOXA7, the closest paralog to HOXB7, was tested first by ONP assays. Similar to the data obtained with HOXB7, HOXA7 DNA-binding affinity was suppressed by overexpression of PARP-1, and partially restored by addition of the PARP inhibitor, DPQ (Fig. 6D). Intriguingly, the transcriptional activity of neither the three HOX proteins that contain Glu-rich tails (HOXB6, HOXC6 and HOXC8), nor the two HOX proteins that lack Glu-rich tails (HOXA1 and HOXA5) were suppressed by overexpression of PARP-1 (Fig. 6E, HOXC8 data not shown). To further understand the determinants of PARP-1 action, we carefully examined the Glu-rich tail of each of these HOX proteins whose activity was not suppressed by PARP-1. We found that the Glu-rich tails in HOXB6, HOXC6 and HOXC8 are disrupted by insertions of basic amino acids lysine and/or arginine, while both HOXA7 and HOXB7 lack such insertions. To test whether the acidic milieu created by uninterrupted stretch of Glu is important for the negative regulation by PARP-1, a HOXB6 mutant form, HOXB6K221E, was created by replacement of the Lys at 221 with Glu. The ONP assay showed that, unlike wild type HOXB6, the DNA binding activity of HOXB6K221E was suppressed by PARP-1 (Fig. 6F). Thus, our data indicated that the uninterrupted glutamic acid stretch in the tails of HOXB7 and HOXA7 was an important determinant for the suppressive modulation by PARP-1.

## Discussion

It is widely acknowledged that members of the HOX gene family function as crucial transcriptional regulators in diverse developmental programs. Recently, a growing body of evidence has also implicated deregulated HOX gene function in various cancers including both leukemia and solid tumors [2], [34]. In stark contrast to their complicated regulatory roles in both physiological and pathological conditions, the consensus core recognition sequence of HOX proteins in DNA is very simple (TAAT/ATTA). The specificity of HOX proteins in transcriptional regulation is mediated through interactions with cofactors [10] that can alter the DNA-binding ability of HOX proteins and increase regulatory specificity. Our previous work showed that HOXB7 binds to components of the DNA repair complex, DNA-PKcs, Ku70/Ku80, and to PARP-1 [17]. In this paper we provide detailed analysis of the binding of HOXB7 to PARP-1, and show that upon binding to PARP-1, HOXB7 undergoes poly(ADP-ribosyl)ation resulting in a reduction of its transcriptional activity

A number of transcriptional factors and chromatin-associated proteins have been reported as binding partners of PARP-1. Interaction with PARP-1 can either increase or decrease the transcriptional activity of its binding partners, frequently through ADP-ribosylation. For instance, PARP-1 acts as a co-activator when associated with ER-alpha, HIF1 and B-MYB [21], [23], [26]. On the other hand, PARP-1 can suppress the transcriptional activity of its partners such as SRY, YY1 and TBP [24], [29], [31] by transferring poly(ADP-ribose) to them. It has been speculated that the high negative charge of the poly(ADP-ribose) reduces the interaction between the DNA-binding elements and transcriptional factors, so that their transcriptional activity is suppressed. In line with this notion, our studies revealed that HOXB7 was poly(ADP-ribosyl)ated by PARP-1 (Fig. 3B, C), and as a result of the interaction and modification, both the transcriptional activity and DNA-binding ability of HOXB7 were repressed (Fig. 4). Furthermore, as seen in Figure 4D and 4F, inhibition of PARP enzymatic activity restored HOXB7 transactivation, which indicated that it was not the interaction per se, but the poly(ADP-ribosyl)ation in particular, that repressed HOXB7 activity. More importantly, HOXB7 transcriptional activity was elevated by suppression of endogenous PARP-1 enzymatic activity either pharmacologically with DPQ or by suppression of PARP-1 expression with specific siRNA (Fig. 4D, E). This suggested that physiologically, PARP-1 functions as a specific modulator to restrict the transcriptional potential of HOXB7. We previously reported that HOXB7 could bind to DNA-PK and enhance its enzymatic activity [17]. Similarly, a recent report described the binding of PARP1 with ERK, which resulted in the activation PARP1 [35]. In contrast, as seen in Fig. 3A, in this study we found that when HOXB7 binds to PARP-1, its enzymatic activity remained unchanged.

Previous studies have reported that the N-terminal octapeptide region [36], [37] and the pentapeptide region [38] in HOXB7, and the automodification domain [24], [39] in PARP-1 are the regions that mediate interactions with their respective partners. However, our analysis has shown that it is the homeodomain in HOXB7 and the first zinc finger domain in PARP-1 that are both DNA-binding domains mediating the interaction. Previous reports have also shown that homeodomain could mediate protein-protein interactions. For instance, the homeodomain of PBX, OCT1 and HOX proteins can mediate interactions with the pentapeptide region in HOX proteins [40], VP16 [41], and CBP [37], respectively. More recently, multiple HOX proteins, including HOXB7, have been reported to interact with SMAD proteins as well through their homeodomain [15]. Similarly, zinc finger domains of PARP-1 have also been demonstrated to interact with SRY [31] or Kruppel-like factor 5 [42]. As discussed above, the physical interaction alone between PARP-1 and the homeodomain in HOXB7 does not affect HOXB7 binding to its consensus binding sites on DNA (Fig. 4C). It appears that the interaction of the two proteins allows the transfer of poly(ADP-ribose) to HOXB7. As shown in Figure 4F, similar to the PARP-1 catalytic mutation E988A, deletion of first zinc finger of PARP-1 also abolished the PARP-1-mediated suppression of HOXB7 transcriptional activity.

Our data also showed that C-terminal Glu-rich tail of HOXB7, which is evolutionarily conserved from zebra fish to humans, is pivotal for the transcriptional suppression of HOXB7 by PARP-1 (Fig. 5). Since the suppression is poly(ADP-ribosyl)ation dependent, and aspartic acid (Asp) and glutamic acid (Glu) residues are acceptors of first ADP-ribose moiety transferred by PARP-1, we reasoned that the Glu-rich tail would be the major site for ADP-ribosylation by PARP-1. The deletion of C-terminal Glu tail significantly reduced the ADP-ribosylation on HOXB7, but not completely abolished the modification (Fig. 5A). This confirms that the Glu-rich tail is the major site but not the only site ADP-ribosylated by PARP-1. Indeed, in addition to the last 7 glutamic acids in the Glu-rich tail, there are 11-Glu and 5-Asp residues scattered in the amino acid sequence of HOXB7 that are potential ADP-ribosylation sites. Importantly, our study showed that the HOXB7 lacking the Glu-rich tail had higher transactivation ability than wild type HOXB7 (Fig. 5D). This finding suggests that the Glu-rich tail functions as an intrinsic negative element in HOXB7, and PARP-1 may possibly regulate the repressive efficacy of the Glu-rich tail by the means of ADP-ribosylation. This observation of the suppressive function of the Glu-rich tail also challenges some published findings. For instance, in one study, deletion of the last 12 amino acids from the C-terminal end dramatically reduced the transcriptional activity of HOXB7 [37]. However, this deletion removed not just the Glu-rich tail, but also 4 additional amino acids upstream of the Glu-rich tail that contain a consensus CKII phosphorylation site, T204 [30]. Based on our data, we speculate that HOXB7’s C-terminal peptides downstream of the homeodomain may act as machinery that precisely fine-tunes the activity of HOXB7. The Glu-rich tail may function negatively, and the CKII target sequence may behave positively in regulation of the transcriptional activity of HOXB7.

Since the interaction domain (the homeodomain) is conserved in HOX proteins, the question arises whether regulation of transcription by all HOX proteins is modulated by PARP-1. If so, how critical is the Glu-rich tail for this interaction? As a matter of fact, all six HOX proteins tested, irrespective of the presence or absence of Glu-rich tails, could interact with PARP-1 and were poly(ADP-ribosyl)ated (Fig. 6A, B). However, we found that this modification suppressed DNA-binding activity in only HOXA7 and HOXB7. These two HOX proteins, unlike the other 3 HOX proteins tested (HOXB6, HOXC6 and HOXC8) that also have Glu-rich tails, are unique in that both have a continuous stretch of acidic C-terminal Glu acid residues. In the others, the acidic environment of Glu residues is interrupted by the insertion of basic amino acids like Lysine and/or Arginine. The importance of the continuous stretch of acidic Glu residues was confirmed in an ONP assays using the mutant, HOXB6K221E (Fig. 6F). The substitution by E for a K at position 221 in the HOXB6 protein created a consistent acidic environment, rendering it sensitive to the suppression by PARP-1.

In summary, this study identifies PARP-1 as a novel partner for HOX proteins, and characterizes the nature of interaction between them. Our study also revealed that all tested HOX proteins were subject to poly(ADP-ribosyl)ation by PARP-1, but transcriptional activity of only HOXA7 and HOXB7 was suppressed by this modification. In this study, we have focused on the influence of the interactions between HOX proteins and PARP-1 on the DNA binding ability of HOX proteins. Whether PARP-1/HOX complexes could affect interactions between HOX proteins and their co-factors, and thereby modulate DNA binding specificity remains to be studied. Based on the evidence that PARP-1-modulates HOX functions, and considering the pivotal role of HOX proteins in physiological and pathological processes, this study suggests a wide potential influence of PARP-1 as well in the regulation of embryonic development and tumorigenesis. Future studies aimed at understanding, in detail, how PARP-1 regulates the binding specificity of HOXB7, HOXA7 and other HOX proteins are needed.

## Materials and Methods

### Cell Culture, Plasmids, Transfection, Antibodies and Other Reagents

All cell lines were obtained from ATCC and cultured as follows: SKBR3 cells in McCoy’s 5A medium containing 15% fetal bovine serum (FBS), and MCF-7 cells, CHO and HeLa cells in DMEM supplemented with 10% FBS. Expression vectors for FLAG-tagged HOXB7 and mutants described in [30] were gifted by Dr. Judith Gasson. Constructs (HOXB7-D1 and HOXB7-D2) containing HOXB7-C-terminal truncations used for *in vitro* transcription and translation (TNT) were generated by PCR and subcloned into the pSG5 vector (Stratagene). Myc-tagged PARP-1 full-length and mutants were gifted by Dr. Perry Kannan [43], and Myc-tagged full-length PARP-1 was then subcloned into pcDNA3.1-Hyg (Invitrogen). The PARP-1 catalytic mutant, PARPE988A, was provided by Dr. Michael Rosenfeld, and subcloned into the pcDNA6 vector. Other PARP-1 deletion constructs including PARP-1-214, PARP-1-372ΔZF1, PARP-1-372ΔZF2, PARPΔZF1, PARPΔZF2 and PARP-NonZF (illustrated in Figure 1D, S1B) were generated by PCR and subcloned into the pcDNA6.0 vector. HOXA5 and HOXA1 were cloned as described [9], [44] and subcloned into pcDNA3.1 vector and fused with an N-terminal FLAG-tag. HOXA7, HOXB6, HOXC6 and HOXC8 were amplified by PCR from IMAGE clones from Open Biosystems (4367439, 2107026, 30915460 and 6156539) and subcloned into pcDNA3.1 vector, in frame with N-terminal FLAG-tag sequence. The TTAT-pGL3 luciferase report plasmid was kindly gifted by Dr. Mark Kamps. All subcloned plasmids were sequenced for verification. Transfections were performed using Lipofectamine 2000 (Invitrogen). The following monoclonal antibodies were used for protein detection by immunoblot: Anti-FLAG M2 (Sigma), anti-PARP monoclonal antibody (clone C-2–10, Invitrogen), polyclonal anti-HOXB7 antibody (Invitrogen) and monoclonal anti-HOXB7 antibody (Abcam). PARP-1 inhibitor, DPQ, was purchased from EMD, ^35^S-MET, ^32^P-ATP, ^32^P-NAD+ from GE Healthcare, and PARG from Biomol.

### Co-immunoprecipitation and GST Pull-down Assay

For co-immunoprecipitation of HOXB7-binding proteins from SKBR3 cells stably expressing HOXB7-YFP or FLAG-tagged HOXB7, 0.5 to 1 mg of cell protein extracts prepared as described in [17], were precleared and subjected to immunoprecipitation for 2.5 h at 4°C with the following antibodies: anti-GFP polyclonal antibodies (Clontech) for immunoprecipitation of HOXB7-YFP, or anti-FLAG polyclonal antibodies (Sigma) for precipitation of FLAG-HOXB7 complexes according to manufacturer’s instructions. To map the domain of HOXB7 interacting with PARP1, myc-tagged PARP1 and FLAG-tagged HOXB7 wild type or its deletion constructs were cotransfected into HEK293T cells. Co-immunoprecipitation was performed with anti-FLAG antibody (Sigma) and western blotted with anti-myc-tag antibody (Millipore) or HRP conjugated FLAG-tag antibody (Sigma). To verify the interaction between endogenous HOXB7 with PARP-1 under physiological conditions, 2 mg of whole cell lysate of MCF-7 cells was subjected to immunoprecipitation with PARP-1 (Invitrogen) or HOXB7 antibodies (Abcam), the immune complexes were loaded onto 4–12% NuPAGE gels (Invitrogen) and immunoblotted with PARP-1 monoclonal (Invitrogen), or anti-HOXB7 rabbit polyclonal antibodies (Invitrogen).

*In vitro* binding experiments were performed by using PARP-1 antibody to immunoprecipitate PARP-1 with full-length HOXB7 and its C-terminal truncations, B7-D1 and B7-D2 from *in vitro* transcription and translation (TNT)-generated proteins, according to the manufacturer’s instructions (Promega). All HOXB7 related proteins were labeled with ^35^S-Met, and the precipitated complex was separated by SDS-PAGE and scanned with a phosphoimager system (Typhoon, GE Healthcare Life Sciences).

GST pull-down assay was performed as described with minor modifications [17]. GST or GST-HOXB7 fusion proteins were generated by transformation of BL21 competent cells with corresponding plasmids and induced with 100 mM IPTG at 30°C for 8 hours. BL21 cells were lysed by sonication, and GST fusion proteins were immobilized on glutathione-Sepharose and incubated with the ^35^S labeled PARP-1 or PARP-1 deletion proteins generated from *in vitro*-TNT in NETN buffer at 4°C for 2 hours. The binding protein complexes were washed 3 times with NETN buffer and subjected to SDS-PAGE and autoradiography. All the immunoprecipitation and GST pull-down assays were repeated at least two times.

### PARP Activity and Poly(ADP-ribosyl)ation Assay

The PARP activity assay was performed as described [45] with some modification. SKBR3 cells were isolated 36 hours after transfection of FLAG-tagged HOXB7 in the absence or presence of DPQ (10 µM). The pellet was incubated with assay buffer containing 50 mM Tris·HCl (pH 8.0), 28 mM KCl, 10 mM MgCl2, 0.01% digitonin in ethanol, 1 mM DTT, and [alpha –^32^P]NAD+ [0.1 µCi/nmol (1 Ci  = 37 GBq)] for 20 min at 4°C. Cells were washed 2 times with assay buffer without ^32^P-NAD. The pellet was lysed and separated by 4–12% SDS-PAGE gel and transferred to nitrocellulose membranes. PARP activity was visualized by PhosphorImager scanning.

The poly(ADP ribosyl)ation assay was performed to detect modification of HOX proteins by PARP-1.4 µg of each of HOX or HOX mutant expression plasmid DNA was transfected into SKBR3 cells, followed by poly(ADP ribosy)lation performed as described above. Cells were then subjected to immunoprecipitation with anti-FLAG antibody and the precipitated proteins were separated by 4–12% SDS-PAGE gel and transferred to nitrocellulose membranes. The membranes were scanned by PhosphorImager system and subsequently used for western blot to determine the levels of the precipitated HOX proteins.

### Luciferase Promoter Reporter Assay

1.5×10^5^ HeLa cells were seeded onto each well of a 24-well plate 24 h prior to transfection. 1–1.5 µg of plasmid DNA was transfected into cells using Lipofectamine 2000 (Stratagene) according to the manufacturer's instructions. 24 hours post transfection cells were harvested for luciferase and β-galactosidase assay using the luciferase activity measuring kit (Promega) and the β-galactosidase assay kit (ICN Biomedicals) according to the manufacturers' instructions. The luciferase activity was normalized to the β-galactosidase activities for each sample. The fold activation was calculated as the ratio of normalized luciferase activity in the cells transfected with the TTAT reporter plasmid in the presence of HOXB7, PBX1 and/or PARP-1 expression plasmid to that in the absence of HOXB7 expression plasmid. Each transfection was repeated at least three times, and means, +/− SD are shown in the figures.

### Knock-down of PARP Expression by siRNA

siRNA oligomer targeted to PARP-1 was purchased from Qiagen (SI02662996). To test the efficiency of the silencing, 100 pmol dsRNA were transfected into 6×10^6^ HeLa cells seeded onto each well of a 6-well plate with Lipofectamine 2000 (Invitrogen) according to the manufacturer’s instructions. 48-hour later, cells were harvested and subjected to western blot. For luciferase promoter activity assay, 20 pmol dsRNA were co-transfected into HeLa cells with the TTATpGL3 luciferase reporter plasmid and different sets of expression vectors as indicated in the figures. Luciferase activity was measured 36 hours post-transfection as described above.

### Electrophoretic Mobility Shift Assay

The radiolabeled probe of HOXB7, oligonucleotides 5′-AAATATCAATTAAATCTTAATTATAA-3′ and 5′-TTATAATTAAGATTTAATTGATATTT-3′ [32] were annealed and labeled with [a-32P]ATP (NEN) and T4 DNA polynucleotide kinase (New England Biolab). Purified PARP-1 (Trevigen, 300 ng), GST-HOXB7 (100 ng), and the ^32^P-labeled probe was incubated in the binding buffer [50 mM Tris-HCl (pH 7.5), 50 mM NaCl, 1 mM MgCl2, 0.5 mM dithiothreitol, 4% glycerol, 0.5 mM EDTA, and 0.25 mg/ml poly(dI-dC)•poly(dI-dC)]. In some reactions, NAD+ (Sigma) was included. After 20 min incubation, samples were separated at room temperature in a native 6% polyacrylamide TBE gel (Invitrogen) at 100 V for 1 hr.

### Oligonucleotide Precipitation Assay (ONP)

The assay was done as previously reported with minor modifications [46]. The nucleotide sequences of biotinylated HOX binding sequence (HBS) were bio-AAATATCAATTAAATCTTAATTATAA and TTATAATTAAGATTTAATTGATATTT [32]. These two complementary strands were annealed in TEN buffer. After transfection with expression plasmids, the SKBR3 cells were lysed by IP buffer (2% Triton-X100, 300 mM NaCl, 20 mM Tris [pH 7.4], 2 mM EDTA, 1% NP40, 1×protease inhibitor cocktail). The cellular debris was removed by centrifugation. Binding buffer (60 mM KCl, 12 mM HEPES [pH 7.9], 4 mM Tris-HCl, 5% glycerol, 0.5 mM EDTA, 1 mM dithiothreitol, 1× protease inhibitor cocktail) was added into the cell extracts (500 µg) to a final volume of 1 ml, and the samples were precleared with 30 µL immobilized streptavidin-agarose beads for 1 hour at 4°C with gentle agitation. The cleared extracts were then incubated with 50 pmol of biotinylated double-strand HBS for 3 hours at 4°C. HBS-bound protein was pulled down by incubating the samples with 20 µL of streptavidin-agarose beads for 1 hour at 4°C with gentle agitation. The precipitated mixture was collected by centrifugation and washed 3 times with cold binding buffer. The samples were subjected to SDS-PAGE and Western blotting for HOX proteins with anti-FLAG antibody. Each ONP assay was performed at least twice, and representative results are presented.

## References

[1] Krumlauf R (1994). Hox genes in vertebrate development.. Cell.

[2] Abate-Shen C (2002). Deregulated homeobox gene expression in cancer: cause or consequence?. Nat Rev Cancer.

[3] Iwasaki M, Kuwata T, Yamazaki Y, Jenkins NA, Copeland NG (2005). Identification of cooperative genes for NUP98-HOXA9 in myeloid leukemogenesis using a mouse model.. Blood.

[4] Kroon E, Krosl J, Thorsteinsdottir U, Baban S, Buchberg AM (1998). Hoxa9 transforms primary bone marrow cells through specific collaboration with Meis1a but not Pbx1b.. EMBO J.

[5] Wu X, Chen H, Parker B, Rubin E, Zhu T (2006). HOXB7, a homeodomain protein, is overexpressed in breast cancer and confers epithelial-mesenchymal transition.. Cancer Res.

[6] Ma XJ, Hilsenbeck SG, Wang W, Ding L, Sgroi DC (2006). The HOXB13:IL17BR expression index is a prognostic factor in early-stage breast cancer.. J Clin Oncol.

[7] Ma XJ, Wang Z, Ryan PD, Isakoff SJ, Barmettler A (2004). A two-gene expression ratio predicts clinical outcome in breast cancer patients treated with tamoxifen.. Cancer Cell.

[8] Chen H, Chung S, Sukumar S (2004). HOXA5-induced apoptosis in breast cancer cells is mediated by caspases 2 and 8.. Mol Cell Biol.

[9] Raman V, Martensen SA, Reisman D, Evron E, Odenwald WF (2000). Compromised HOXA5 function can limit p53 expression in human breast tumours.. Nature.

[10] Mann RS, Affolter M (1998). Hox proteins meet more partners.. Curr Opin Genet Dev.

[11] Lu Q, Kamps MP (1997). Heterodimerization of Hox proteins with Pbx1 and oncoprotein E2a-Pbx1 generates unique DNA-binding specifities at nucleotides predicted to contact the N-terminal arm of the Hox homeodomain–demonstration of Hox-dependent targeting of E2a-Pbx1 in vivo.. Oncogene.

[12] Chang CP, Shen WF, Rozenfeld S, Lawrence HJ, Largman C (1995). Pbx proteins display hexapeptide-dependent cooperative DNA binding with a subset of Hox proteins.. Genes Dev.

[13] Lu Q, Knoepfler PS, Scheele J, Wright DD, Kamps MP (1995). Both Pbx1 and E2A-Pbx1 bind the DNA motif ATCAATCAA cooperatively with the products of multiple murine Hox genes, some of which are themselves oncogenes.. Mol Cell Biol.

[14] Shen WF, Krishnan K, Lawrence HJ, Largman C (2001). The HOX homeodomain proteins block CBP histone acetyltransferase activity.. Mol Cell Biol.

[15] Li X, Nie S, Chang C, Qiu T, Cao X (2006). Smads oppose Hox transcriptional activities.. Exp Cell Res.

[16] Williams TM, Williams ME, Heaton JH, Gelehrter TD, Innis JW (2005). Group 13 HOX proteins interact with the MH2 domain of R-Smads and modulate Smad transcriptional activation functions independent of HOX DNA-binding capability.. Nucleic Acids Res.

[17] Rubin E, Wu X, Zhu T, Cheung JC, Chen H (2007). A role for the HOXB7 homeodomain protein in DNA repair.. Cancer Res.

[18] Jagtap P, Szabo C (2005). Poly(ADP-ribose) polymerase and the therapeutic effects of its inhibitors.. Nat Rev Drug Discov.

[19] Kim MY, Zhang T, Kraus WL (2005). Poly(ADP-ribosyl)ation by PARP-1: ‘PAR-laying’ NAD+ into a nuclear signal.. Genes Dev.

[20] Cervellera MN, Sala A (2000). Poly(ADP-ribose) polymerase is a B-MYB coactivator.. J Biol Chem.

[21] Santilli G, Cervellera MN, Johnson TK, Lewis RE, Iacobelli S (2001). PARP co-activates B-MYB through enhanced phosphorylation at cyclin/cdk2 sites.. Oncogene.

[22] Anderson MG, Scoggin KE, Simbulan-Rosenthal CM, Steadman JA (2000). Identification of poly(ADP-ribose) polymerase as a transcriptional coactivator of the human T-cell leukemia virus type 1 Tax protein.. J Virol.

[23] Elser M, Borsig L, Hassa PO, Erener S, Messner S (2008). Poly(ADP-ribose) polymerase 1 promotes tumor cell survival by coactivating hypoxia-inducible factor-1-dependent gene expression.. Mol Cancer Res.

[24] Oei SL, Griesenbeck J, Schweiger M, Babich V, Kropotov A (1997). Interaction of the transcription factor YY1 with human poly(ADP-ribosyl) transferase.. Biochem Biophys Res Commun.

[25] Butler AJ, Ordahl CP (1999). Poly(ADP-ribose) polymerase binds with transcription enhancer factor 1 to MCAT1 elements to regulate muscle-specific transcription.. Mol Cell Biol.

[26] Ju BG, Lunyak VV, Perissi V, Garcia-Bassets I, Rose DW (2006). A topoisomerase IIbeta-mediated dsDNA break required for regulated transcription.. Science.

[27] Nirodi C, NagDas S, Gygi SP, Olson G, Aebersold R (2001). A role for poly(ADP-ribose) polymerase in the transcriptional regulation of the melanoma growth stimulatory activity (CXCL1) gene expression.. J Biol Chem.

[28] Pavri R, Lewis B, Kim TK, Dilworth FJ, Erdjument-Bromage H (2005). PARP-1 determines specificity in a retinoid signaling pathway via direct modulation of mediator.. Mol Cell.

[29] Kraus WL, Lis JT (2003). PARP goes transcription.. Cell.

[30] Yaron Y, McAdara JK, Lynch M, Hughes E, Gasson JC (2001). Identification of novel functional regions important for the activity of HOXB7 in mammalian cells.. J Immunol.

[31] Li Y, Oh HJ, Lau YF (2006). The poly(ADP-ribose) polymerase 1 interacts with Sry and modulates its biological functions.. Mol Cell Endocrinol.

[32] Chen H, Rubin E, Zhang H, Chung S, Jie CC (2005). Identification of transcriptional targets of HOXA5.. J Biol Chem.

[33] Marsischky GT, Wilson BA, Collier RJ (1995). Role of glutamic acid 988 of human poly-ADP-ribose polymerase in polymer formation. Evidence for active site similarities to the ADP-ribosylating toxins.. J Biol Chem.

[34] Chen H, Sukumar S (2003). HOX genes: emerging stars in cancer.. Cancer Biol Ther.

[35] Cohen-Armon M, Visochek L, Rozensal D, Kalal A, Geistrikh I (2007). DNA-independent PARP-1 activation by phosphorylated ERK2 increases Elk1 activity: a link to histone acetylation.. Molecular cell.

[36] Chariot A, Princen F, Gielen J, Merville MP, Franzoso G (1999). IkappaB-alpha enhances transactivation by the HOXB7 homeodomain-containing protein.. J Biol Chem.

[37] Chariot A, van Lint C, Chapelier M, Gielen J, Merville MP (1999). CBP and histone deacetylase inhibition enhance the transactivation potential of the HOXB7 homeodomain-containing protein.. Oncogene.

[38] Knoepfler PS, Kamps MP (1995). The pentapeptide motif of Hox proteins is required for cooperative DNA binding with Pbx1, physically contacts Pbx1, and enhances DNA binding by Pbx1.. Mol Cell Biol.

[39] Simbulan-Rosenthal CM, Rosenthal DS, Luo R, Samara R, Espinoza LA (2003). PARP-1 binds E2F-1 independently of its DNA binding and catalytic domains, and acts as a novel coactivator of E2F-1-mediated transcription during re-entry of quiescent cells into S phase.. Oncogene.

[40] Chang CP, Shen WF, Rozenfeld S, Lawrence HJ, Largman C (1995). Pbx proteins display hexapeptide-dependent cooperative DNA binding with a subset of Hox proteins.. Genes & development.

[41] Stern S, Tanaka M, Herr W (1989). The Oct-1 homoeodomain directs formation of a multiprotein-DNA complex with the HSV transactivator VP16.. Nature.

[42] Suzuki T, Nishi T, Nagino T, Sasaki K, Aizawa K (2007). Functional interaction between the transcription factor Kruppel-like factor 5 and poly(ADP-ribose) polymerase-1 in cardiovascular apoptosis.. J Biol Chem.

[43] Li M, Naidu P, Yu Y, Berger NA, Kannan P (2004). Dual regulation of AP-2alpha transcriptional activation by poly(ADP-ribose) polymerase-1.. Biochem J.

[44] Zhang X, Zhu T, Chen Y, Mertani HC, Lee KO (2003). Human growth hormone-regulated HOXA1 is a human mammary epithelial oncogene.. J Biol Chem.

[45] Ha HC, Hester LD, Snyder SH (2002). Poly(ADP-ribose) polymerase-1 dependence of stress-induced transcription factors and associated gene expression in glia.. Proc Natl Acad Sci U S A.

[46] Zhang S, Fei T, Zhang L, Zhang R, Chen F (2007). Smad7 antagonizes transforming growth factor beta signaling in the nucleus by interfering with functional Smad-DNA complex formation.. Mol Cell Biol.

